# Scale‐dependent effects of neighborhood biodiversity on individual tree productivity in a coniferous and broad‐leaved mixed forest in China

**DOI:** 10.1002/ece3.6530

**Published:** 2020-07-08

**Authors:** Chunyu Fan, Lingzhao Tan, Chunyu Zhang, Xiuhai Zhao, Lushuang Gao, Klaus von Gadow

**Affiliations:** ^1^ Research Center of Forest Management Engineering of State Forestry and Grassland Administration Beijing Forestry University Beijing China; ^2^ Faculty of Forestry and Forest Ecology Georg‐August‐University Göttingen Göttingen Germany; ^3^ Department of Forest and Wood Science University of Stellenbosch Stellenbosch South Africa

**Keywords:** biodiversity‐productivity relationships, competition, individual perspective, niche complementary, scale dependence

## Abstract

The relationship between biodiversity and productivity has stimulated an increasing body of research over the past decades, and this topic still occupies a central place in ecology. While most studies have focused on biomass production in quadrats or plots, few have investigated the scale‐dependent relationship from an individual plant perspective. We present an analysis of the effects of biodiversity (species diversity and functional diversity) on individual tree growth with a data set of 16,060 growth records from a 30‐ha temperate forest plot using spatially explicit individual tree‐based methods. A significant relationship between species diversity and tree growth was found at the individual tree level in our study. The magnitude and direction of biodiversity effects varies with the spatial scale. We found positive effects of species diversity on tree growth at scales exceeding 9 m. Individual tree growth rates increased when there was a greater diversity of species in the neighborhood of the focal tree, which provides evidence of a niche complementarity effect. At small scales (3–5 m), species diversity had negative effects on tree growth, suggesting that competition is more prevalent than complementarity or facilitation in these close neighborhoods. The results also revealed many confounding factors which influence tree growth, such as elevation and available sun light. We conclude that the use of individual tree‐based methods may lead to a better understanding of the biodiversity‐productivity relationship in forest communities.

## Graphical abstract

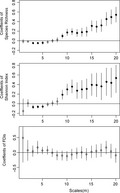

## 1 INTRODUCTION

During the past three decades, the influence of biodiversity on ecosystem functioning, especially on biomass productivity, has aroused considerable interest among ecologists (Cardinale et al., 2012; Gadow, Zhang, Durrheim, Drew, & Armin Seydack, 2016; Hooper et al., 2005; Liang, Zhou, Tobin, McGuire, & Reich, 2015; Loreau & Hector, 2001; Wang et al., 2019). Many studies have revealed a positive effect of biodiversity on productivity (Gamfeldt et al., 2013; Liang et al., 2015; Paquette & Messier, 2011) using forest inventory data across the world. Two hypotheses have been proposed to explain this result: the complementarity effect hypothesis and the sampling effect hypothesis (Forrester & Bauhus, 2016; Thompson, Mackey, McNulty, & Mosseler, 2009). The complementarity effect hypothesis which is the result of niche partitioning or facilitation proposes that communities with more species are able to access and use limited resources more efficiently (Cardinale et al., 2007; Loreau, 1998; Tilman, 1997). The sampling effect hypothesis claims that communities with higher diversity are more productive because they have a higher probability of containing at least one species that is very productive, which highlights the role of dominant species (Huston, 1997; Nguyen, Herbohn, & Firn, 2012).

Despite detailed studies, the relationship between biodiversity and productivity remains controversial, especially in natural forest ecosystems. Besides positive relationships, negative and neutral relationships have also been reported (Adler et al., 2011; Waide et al., 1999). The variability of this relationship may be caused by differences related to management and other disturbances, forest age, and soil or light conditions (Forrester & Bauhus, 2016; Pretzsch et al., 2013; Vilà, Vayreda, Gracia, & Ibáñez, 2003). In addition, methodological differences between individual studies such as the choice of diversity index, the chosen level (quadrat or individual tree), and spatial scales (local or regional) of specific study could also lead to inconsistent conclusions (Huston, 1997; Loreau, Mouquet, & Gonzalez, 2003; Padilla‐Martínez et al., 2020; Schulze & Mooney, 1993).

There is no consensus regarding the appropriate measure of biodiversity in the study of the biodiversity‐productivity relationship. Species diversity indices were mostly used in the past, but recent studies have shown that the use of species diversity indices may disregard some of the functional dissimilarities between species, which can lead to inconsistent assessments of biodiversity (Hao, Zhang, Zhao, & von Gadow, 2018; Laliberté & Legendre, 2010). Functional traits are important for plant growth because of the potential relationships between traits and niche occupancy or partitioning. Trait similarities can be used as surrogates for niche similarity (Chen, Wright, Muller‐Landau, Wang, & Yu, 2016). An increasing degree of diversification of functional traits within tree neighborhoods may lead to increasing productivity of individual trees due to niche complementarity (Fichtner et al., 2017; Forrester & Bauhus, 2016). Many functional diversity indices have been introduced to describe the distribution of functional traits in a community of interest. Including functional diversity may provide more effective links between biodiversity and productivity than mere species‐based diversity.

Most studies of the biodiversity‐productivity relationship in natural forests have used quadrat‐based methods in the analyses (Chisholm et al., 2013; Ruiz‐Benito et al., 2014). Although quadrat‐based methods provide a direct characterization of the shape of the relationship, the use of quadrats as the unit of study inevitably neglected interactions at smaller spatial scales, such as facilitation and competition. For example, niche complementarity is expected to be restricted to interactions among close neighbors (because trees are sessile; see Weiner, 1990). To overcome this limitation, several recent studies have replaced quadrat‐based methods with individual tree‐based methods (Chen et al., 2016; Fichtner et al., 2018; Fien, Fraver, Teets, Weiskittel, & Hollinger, 2019; Lasky, Uriarte, Boukili, & Chazdon, 2014; Ramage et al., 2017; Uriarte, Condit, Canham, & Hubbell, 2004; Uriarte et al., 2010; Vitali, Forrester, & Bauhus, 2018). The interaction among trees is a spatially relevant process, especially in natural forest with complex structure and species composition (D’Amato & Puettmann, 2004). The individual tree‐based methods can explicitly incorporate the spatial structure of the local neighborhood and are thus more realistic. Scaling down to individual tree level processes can advance our understanding of the mechanisms underlying biodiversity‐productivity relationships.

Relationships between biodiversity and productivity have been shown to be scale‐dependent at community or quadrat‐level (Chisholm et al., 2013; Luo, Liang, Cazzolla Gatti, Zhao, & Zhang, 2019). Previous studies reporting biodiversity effects on individual tree growth in mixed‐species forests often involved only one spatial scale (Ratcliffe, Holzwarth, Nadrowski, Levick, & Wirth, 2015; von Oheimb et al., 2011). There is increasing evidence that the relative strength of neighborhood interactions (facilitation and competition) in forests may change with the spatial scale (Chen et al., 2016; Fichtner et al., 2017). However, the question remains whether the magnitude and direction of biodiversity effects on individual tree growth varies with increasing neighborhood scale.

Studies have shown that neighborhood interaction could modify the relationship between biodiversity and productivity (Jucker et al., 2014). Interaction among neighbors can have a strong impact on individual tree growth (Lee, Gadow, Chung, & Lee, 2004; Potvin & Dutilleul, 2009). In addition to the competition reduction caused by niche complementarity or facilitation, the increase in heterospecific neighbors may result in neutral or negative effects on tree growth due to competition for limited resource (von Oheimb et al., 2011). The application of individual‐based methods permits more detailed analysis. It is possible to discern whether observed individual tree growth enhancement is driven by altered modes of interaction (competition or facilitation) at different spatial scales.

In addition to neighborhood interactions, abiotic conditions such as topography and radiation are recognized as important factors influencing species diversity and individual tree growth at local scales (Forrester & Bauhus, 2016; Huston, 1999; Sanchez‐Gomez, Zavala, Van Schalkwijk, Urbieta, & Valladares, 2008). Based on a literature review, Hooper et al. (2005) found that environmental conditions can modify complementarity effects in structuring communities. Specific ecosystem properties are often more influenced by abiotic conditions than by species richness (Finegan et al., 2015). Given such confounding influences, the identification of biodiversity effects on tree productivity is inherently context‐dependent. A more differentiated approach by incorporating competition and abiotic factors was recommended by Pretzsch et al. (2013), especially with reference to natural forests.

This study is based on a large set of spatially explicit individual‐based observations. It is therefore possible to investigate the spatial scale dependence of biodiversity effects on individual tree growth while controlling for tree size, abiotic environmental variables, and competition in a conifer and broad‐leaved mixed forest in Northeastern China. The specific objectives are to: (a) investigate the effects of different biodiversity indices (in terms of species or functional diversity) on individual biomass productivity, (b) evaluate the scale dependence of the biodiversity effects within tree neighborhoods, (c) test whether the biodiversity effects are mediated by competition, (d) explore how individual tree growth is affected by abiotic conditions after detecting a significant biodiversity‐productivity relationship.

## 2 MATERIALS AND METHODS

### 2.1 Study area

The study was carried out in a typical temperate mixed broadleaf‐conifer forest (43°51'–44°05'N, 127°35'–127°51'E), which is under the jurisdiction of the Jiaohe Administrative Bureau in Jilin province, northeastern China (Zhao, Corral‐Rivas, Zhang, Temesgen, & Gadow, 2014). The forest is far away from residential areas where human disturbance has been virtually unknown. This area has a temperate continental mountain climate affected by monsoons. The average temperature is −18.6°C during the coldest days in January, and 21.7°C during the hottest days in July. The mean annual rainfall is 606 mm. The soil type is a dark brown forest soil and the rootable depth ranges between 20 and 100 cm. The top five species in basal area are *Ulmus laciniate*, *Acer mono*, *Tilia amurensis*, *Pinus koraiensis*, and *Betula costata*.

### 2.2 Data collection

This study is based on observations collected in an unmanaged forest plot covering 30 ha, which was established in 2010. The first census of the plot was started in August 2010. We tagged and mapped all individual woody stems with DBH ≥ 1 cm, identified each species (Table S1), and measured all diameters at breast height (DBH) and heights. A second census was carried out in August 2015. The status of trees (dead or alive) and the DBH for trees alive were recorded. Individuals showing negative increment had to be discarded because the accuracy of the first measurement could not be assessed (following Chen et al., 2016; Condit, 1998). We only considered individuals as focal trees which had been available at both censuses. Dead trees and recruits were excluded.

### 2.3 Individual tree productivity

All woody plants with a DBH larger than 5 cm in the first census were included in this study. The aboveground biomass (AGB) of each tree was estimated using existing allometric regression equations based on the measurement of tree diameter. A logarithmic model was used to fit allometric relationships between the aboveground woody biomass (AGB) and tree DBH (See Tables S2 and S3). The fitting equation is ln(AGB) = *a* + b*ln(DBH), where AGB is aboveground woody biomass and DBH is the diameter at breast height. The goodness of fit of the allometric model was evaluated using the coefficient of determination (*R* ^2^). The significance of coefficients was calculated for each regression. The fit was evaluated by analyzing the residuals and using root mean square error (RMSE).

There are 29 woody tree species which are included in the study (Table S1). For species without available model, their model parameters are assumed to be valid for species of the same genus or with similar stem form. For trees alive in 2015, we calculated the annual aboveground biomass increment of every focal individual using the following equation:

$${\text{deltaAGB}}_{i}=\frac{{\text{AGB}}_{15i}-{\text{AGB}}_{10i}}{5}$$

where the deltaAGB of *i*th tree is annual increment in aboveground biomass from 2010 to 2015. AGB_15_ *_i_* and AGB_10_ *_i_* represent AGB of that tree in 2015 and 2010, respectively.

### 2.4 Biodiversity measures

#### 2.4.1 Species diversity

Species diversity was calculated using species richness and Shannon index within the neighborhood of a variety of radii (1 m, 2 m, 3 m, …20 m, in steps of 1 m) for each focal tree. Only tree and shrub species were included. Species richness (SR) represents the number of species in the neighborhood. The Shannon index (*H_s_*) which incorporates species richness and evenness was calculated as follows:

$$H_{s}=-\sum_{i=1}^{\text{SR}}\frac{n_{i}}{N}\ln\left(\frac{n_{i}}{N}\right)$$

where $n_{i}$ is the number of individuals of species *i* and *N* is the number of all neighbors within a chosen circle around the focal tree.

#### 2.4.2 Functional diversity

In 2018, functional traits were determined for 29 woody species in our plot. The traits include an architectural trait (maximum height), a wood trait (wood density), and five leaf traits (leaf area, specific leaf area, leaf carbon concentration, leaf nitrogen concentration, and leaf carbon‐nitrogen ratio). We measured maximum height using an altimeter pole and a laser telemeter (TruPulse360, Laser TechnologyInc., USA). For each species, wood and leaf traits were collected from 10 to 30 individuals. Wood cores were extracted from the cortex to the pith at 1.3 m height using an increment borer (5 mm, Suunto, Finland) to determine the wood density, by dividing the wood core dry weight (80°C, 72 hr) by its fresh volume. Leaf traits were measured on individuals with DBH between 10 and 20 cm. We took five fresh leaf samples on the highest parts of the tree crown from each individual. Following the standard methods proposed by Cornelissen et al. (2003), we scanned leaves to obtain a computer image, and measured the leaf area by using the image analysis software *Image J*. Fresh leaf samples were weighed and oven dried at 60°C for at least 72 hr. We weighed the leaf dry mass, then leaf dry matter content (leaf dry mass/leaf fresh mass), and specific leaf area (leaf area/dry matter) were obtained. Leaf carbon and nitrogen concentrations were assessed using an elemental analyzer (PE2400 SeriesII, PerkinElmer Inc., USA). Leaf carbon‐nitrogen ratios were calculated by dividing the leaf carbon concentrations by the leaf nitrogen concentrations (Table S4).

To calculate the functional diversity for multiple traits, we followed the concept of functional trait space based on a geometrical point of view (Cornwell, Schwilk, & Ackerly, 2006; Villéger, Mason, & Mouillot, 2008). If T functional traits values were considered, the functional traits space can be described as a T dimensional space defined by T axes, each one corresponding to a specific trait. For every species of the community, the standardized values of T traits are conceived as coordinates in the functional trait space. All species can thus be located in a multidimensional functional space. FDis (Functional dispersion), as defined by Laliberté and Legendre (2010), has been shown to be a useful functional diversity index. FDis, calculated as the mean distance in a multidimensional trait space of individual species to the centroid of all species in the neighborhood, represents the functional dissimilarity around each focal tree.

### 2.5 Local competition

We calculated the conventional *Hegyi* competition index (Hegyi, 1974):

$$\text{Hegyi}=\sum_{j=1}^{n}\frac{D_{j}}{D_{i}}\cdot \frac{1}{d_{\mathrm{ij}}}$$

*n* is the number of neighbors within the circle of *r* m radius. $D_{i}$ is the diameter of the focal tree, and $D_{j}$ is the diameter of the neighbors. $d_{\mathrm{ij}}$ is the horizontal distance between focal tree *i* and its neighbor *j*.

### 2.6 Neighborhood size structure

We calculated the dominance of each focal tree within its neighborhood (Gadow, 1996; Hui & Gadow, 2002; Ni, Baiketuerhan, Zhang, Zhao, & Gadow, 2014; Staupendahl & Zucchini, 2006). The dominance index reflects the relative dominance of the focal tree within its immediate neighborhood and was calculated as:

$$U_{i}=\frac{1}{n}\sum_{j=1}^{n}k_{\mathrm{ij}},k_{\mathrm{ij}}=\left\{\begin{array}{c} 0,D_{i}<D_{j}\\ 1,\text{otherwise} \end{array}\right.$$

### 2.7 Abiotic variables

We investigated four topographical variables at the 20 m × 20 m scale, that is, slope, aspect, convexity, and mean elevation as a proxy for local topographic condition. The mean elevation is measured as the mean elevation value of the four corners of each quadrat. The elevation ranges from 576 m to 784 m above sea level (Figure S1). The slope is the mean angle of inclination of the four triangular planes formed by any three quadrat corners. Aspect is calculated as the average angle of the four triangular planes that deviate from the north direction, and the values range between 0 and 360°. The convexity of a quadrat was calculated as the elevation of the focal quadrat minus the mean elevation of the eight surrounding quadrats. When a quadrat is located at the plot edge, convexity was taken as the elevation of the center point minus the mean of the four corners.

We used canopy openness of a quadrat as a proxy of the sun light condition for each individual. Canopy openness was determined from hemispherical canopy photographs at the center of each quadrat in August 2012. Images were analyzed using the programs WinSCANOPY and XLScanopy (Yan, Zhang, Wang, Zhao, & Gadow, 2015). Soil samples were collected in each quadrat. Eight soil properties were determined including pH, the amount of organic matter, and the total amounts as well as the available nutrients of nitrogen (N), phosphorus (P), and potassium (K). We performed principal components analysis (PCA) to reduce the number of variables. The first five components explained 81% of the variation in soil conditions (Table S5).

In summary, topographic variables, canopy openness, and soil properties were used as indicators of the abiotic environmental condition for each focal individual.

### 2.8 Statistical analysis

We used a spatially explicit regression model to quantify the empirical relationship between biodiversity and individual tree growth. The following model form proposed by Liang, Crowther, and Picard (2016) was chosen:

$$P=\beta f\left(X\right)B^{\theta }$$

Where *P* represents the individual productivity; *B* is the biodiversity index. *f*(*X*) is a function of the control variables selected from abiotic variables, competition, and neighborhood size structure covariates, and *β* is a vector of coefficients. The elasticity of substitution θ represents the degree to which species can substitute each other in contributing to productivity (Liang et al., 2015, 2016). It can be used to measure the strength of the effect of biodiversity on productivity. The log‐transformed version of the model was applied in this study:

$$\log\left(P\right)=\beta \cdot \log\left(f\left(X\right)\right)+\theta \cdot \log\left(B\right)$$

Since there are many covariates for competition, neighborhood composition, and abiotic condition, we first selected the most significant variables which should be considered in the models. Function “dredge” in the R package “MuMIn” was used to determine the most appropriate and parsimonious model (Table S6). The interactions between biodiversity and competition were added to detect their interactive effects on tree growth. We compared models with and without this interaction term using a likelihood ratio test. After initial investigations, the following model was chosen:

$$\log\left(\text{deltaAGB}\right)=\beta_{0}+\theta \log\left(B\right)+\beta_{1}\log\left(\text{DBH}\right)+\beta_{2}\log\left(\text{canopy openness}\right)+\beta_{3}\log\left(\text{elevation}\right)+\beta_{4}\log\left(U\right)+\beta_{5}\log\left(\text{Hegyi index}\right)+\beta_{6}\log\left(\text{Hegyi index}\right):\log\left(B\right)$$

To test the possible relationships between biodiversity surrogate measures and individual tree growth, we chose one biodiversity index each time when fitting the models (Table 1).

**TABLE 1:** Model description of different best‐fit biodiversity models

Models	Descriptions
Species richness model	SR + DBH+Elevation + Canopy+U + SR:Hegyi
Shannon index model	Hs + DBH+Elevation + Canopy+U + Hs:Hegyi
Functional diversity model	FDis + DBH+Elevation + Canopy+U + Hegyi

A series of models were fitted to detect the scale dependency of biodiversity effects on productivity by setting (1 m, 2 m, … 19 m, 20 m) radii (with 1 m increment) for the neighborhood circle around the focal tree. We fitted the models using “lmer” function in the R package “lme4” (Pinheiro, Bates, DebRoy, Sarkar, & Team, 2013). Spatial autocorrelation was considered by adding a random effect of quadrat in which an individual tree was located. Given the different life‐history strategies, tree growth of different species is expected to respond differently to biodiversity in the neighborhoods. Therefore, we also included species identity as a random effect.

## 3 RESULTS

### 3.1 Description of biodiversity and individual productivity

A total of 16,060 individual trees were alive, both in 2010 and 2015. Comparison of the productivity between species showed that individual tree growth rates varied significantly among species in terms of AGB. The canopy species *Juglans mandshurica*, which is very productive in our study area had a mean AGB increment of 12.6 (3.6–21.6) kg/yr. The individual tree productivity was relatively low for shrub species, such as *Euonymus phellomanus* and *Acer barbinerve*.

**FIGURE 1:**
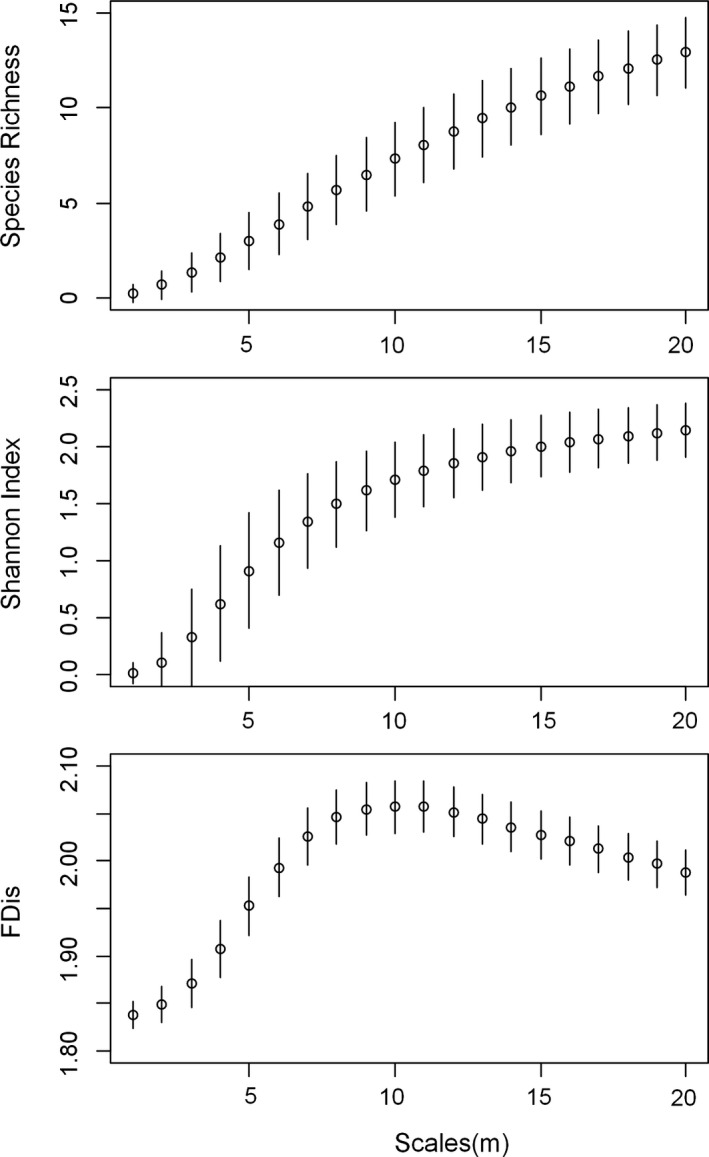
Mean value (dot) and standard deviation (bar) of biodiversity indices at different scales

The mean number of species within the neighborhood radius from 1 m to 20 m for each focal tree ranged from 0.2 to 13.6. The mean value of Shannon index increased with the scale, from 0.1 to 2.14. The functional diversity values were also different among spatial scales: Functional dispersion (FDis) increased with increasing scale at first (Figure 1) and then decreased after the 10 m scale.

### 3.2 Individual‐level analysis

**FIGURE 2:**
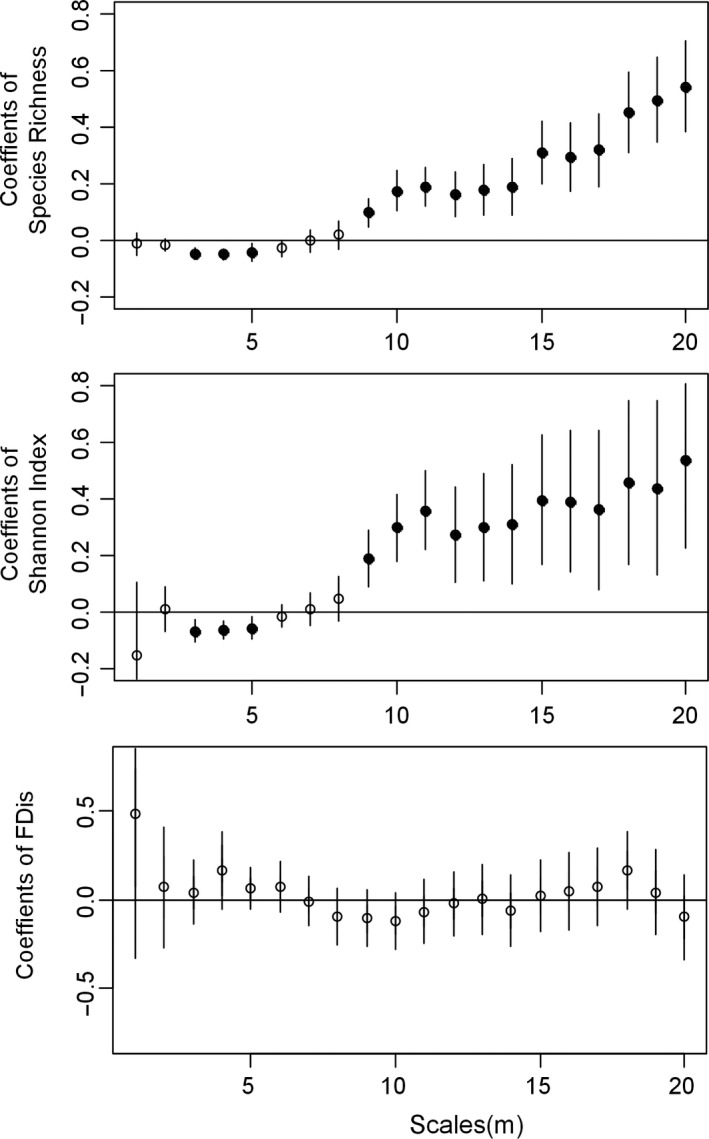
Coefficients of biodiversity indices in the individual tree growth model. The bar for each dot represents the standard deviation. Solid dots represent significant biodiversity effects

The results of the linear mixed effects models showed that biodiversity was significantly correlated with tree growth, but that effect changed with the spatial scale. Species richness and the Shannon index are significantly negatively correlated with tree growth at 3 m to 5 m scales. Positive effects of species diversity were found at scales greater than 9 m. The strength of the positive species diversity effect showed an upward trend. However, the correlation with tree growth of the functional dispersion index (FD_is_) was nonsignificant at all scales (Figure 2).

We found significant effects of the interactions between species diversity indices and competition at the scales from 5 m to 20 m (Tables 2, 3, Tables S7–S14). This is an indication that competition influences the effect of species diversity on tree growth. There is a significant difference between models containing the interactions and those which do not (χ^2^ = 8.9, *p* = .0017). However, this was not the case for the functional diversity model (χ^2^ = 2.0, *p* = .08). The interaction between competition and functional diversity did not show up in the best‐fit functional diversity model (Table 4, Tables S15–S18). As expected, the Hegyi competition index in the functional diversity model was negatively correlated with tree growth.

**TABLE 2:** Estimates of the significant control variables in the best‐fit species richness model at 5, 10, 15, and 20 m scale. The results of other scales were shown in Appendix S1

Control Variables	Coefficients 5 m	Coefficients 10 m	Coefficients 15 m	Coefficients 20 m
DBH	1.66^***^	1.58^***^	1.54^***^	1.52^***^
Dominance index(U)	0.21^***^	0.20^***^	0.21^***^	0.21^***^
Elevation	0.50^***^	0.55^***^	0.56^***^	0.56^***^
Canopy openness	0.17^***^	0.16^***^	0.15^***^	0.15^***^
SR: Hegyi	−0.07^***^	−0.14^***^	−0.15^***^	−0.21^***^

*** represents *p*<.001

**TABLE 3:** Estimates of the significant control variables in the best‐fit Shannon index model at 5, 10, 15, and 20 m scale. The results of other scales were shown in Appendix S1

Control Variables	Coefficients 5 m	Coefficients 10 m	Coefficients 15 m	Coefficients 20 m
DBH	1.66^***^	1.58^***^	1.54^***^	1.52^***^
Dominance index(U)	0.21^***^	0.21^***^	0.21^***^	0.22^***^
Elevation	0.52^***^	0.55^***^	0.55^***^	0.54^***^
Canopy openness	0.17^***^	0.16^***^	0.15^***^	0.15^***^
Hs: Hegyi	−0.11^***^	−0.25^***^	−0.18^**^	−0.19^**^

** represents *p*<.01

*** represents *p*<.001

**TABLE 4:** Estimates of the significant control variables in the best‐fit functional diversity model at 5, 10, 15, and 20 m scale. The results of other scales were shown in Appendix S1

Control Variables	Coefficients 5 m	Coefficients 10 m	Coefficients 15 m	Coefficients 20 m
DBH	1.66^***^	1.58^***^	1.54^***^	1.52^***^
Dominance index(U)	0.21^***^	0.21^***^	0.21^***^	0.22^***^
Elevation	0.56^***^	0.55^***^	0.56^***^	0.54^***^
Canopy openness	0.17^***^	0.16^***^	0.15^***^	0.15^***^
Hegyi index	−0.07^***^	−0.14^***^	−0.17^***^	−0.19^***^

*** represents *p*<.001

As for the control variables, we found consistent results among spatial scales. An expected strong positive relationship between the Dominance of the focal tree and its growth was detected at all scales (Tables 2, 3, 4). Among the topographic variables, the growth of individual trees was positively related to elevation and canopy openness. Other environmental variables such as slope, convexity, and soil characteristics had no impact on tree growth.

## 4 DISCUSSION

In this study, we analyzed a data set of 16,060 growth records from a 30‐ha temperate forest plot to evaluate the biodiversity‐productivity relationship at multiple scales using a spatially explicit individual‐based method. Scientists have highlighted the fundamental role of spatial scale in ecological research (Chisholm et al., 2013; Luo et al., 2019). Previous studies have investigated the effect of biodiversity on growth from an individual tree perspective (Chen et al., 2016; Fichtner et al., 2018; Fien et al., 2019; Lasky et al., 2014; Ramage et al., 2017; Vitali et al., 2018). However, most of them focused only on one neighborhood scale. For example, neighbors were identified within a radius of 30 m and niche complementarity was found in a 50‐ha tropical forest (Chen et al., 2016). Contrary to a previous study which showed that biodiversity had rare or weak effects in species‐rich subtropical forest stands (Von Oheimb et al., 2011), a significant relationship between biodiversity and tree growth was found at the individual level in our study. Meanwhile, the magnitude and direction of biodiversity effects varied with the spatial scale, which is an indication that the neighborhood scale matters when analyzing the relationship between biodiversity and productivity.

The positive effect of species diversity on tree growth at scales greater than 9 m was consistent with a study in temperate forests involving European beech (*Fagus sylvatica*) and Norway spruce (*Picea abies*), which revealed a positive effect of local neighborhood mixing on biomass production (Pretzsch & Schütze, 2009). Our result provided evidence of niche complementarity in temperate forests.

We did not find a strong positive effect of functional diversity on tree growth in this study, which is in line with other studies in tropical (Finegan et al., 2015) and subtropical (Ali, Yan, Chang, Cheng, & Liu, 2017; Gadow et al., 2016) forests that showed no increase in productivity with functional diversity. The positive effects of biodiversity can be partitioned in complementarity and selection effects (Loreau & Hector, 2001). One possible reason for our result is that individual productivity may be more closely related to the trait values of the dominant contributors, thus supporting the selection effects hypothesis. However, this hypothesis could not be confirmed based on our functional dispersion (FD_is_) parameter described in the methods section. An alternative explanation for the lack of a positive effect of functional diversity may be that the trait dataset that we used might not reflect niche complementarity adequately. It seems necessary to consider additional traits in future investigations, such as photosynthetic capacity, and crown plasticity.

At small scales (3–5 m), species diversity had negative effects on tree growth. This result was not surprising because at this scale, competitive exclusion may be the most likely result of species interactions. In mixed‐species forests, competition is a complex phenomenon and an important source of variation in individual tree growth (Gadow, 1996; Lee et al., 2004; Li, Liang, Du, & Ma, 2010; Zhao, Borders, Wilson, & Rathbun, 2006). We detected a positive relationship between species diversity and competition (Figure S2), which suggests that the diversity effect may be mediated by competition to some degree. Based on the data from more than 12,000 permanent forest plots, from temperate to boreal forests, Paquette and Messier (2011) have shown that complementarity may be less important in temperate forests. Similarly, negative relationships have also been found in old‐growth forests as a consequence of increased resource competition (Finegan et al., 2015). These findings support our result, indicating that competition is a key determinant that shapes the outcome of the biodiversity and productivity relationship.

The significant interaction between species diversity and competition index provides further evidence of the necessity to consider competition when analyzing biodiversity‐productivity relationships. Fichtner et al. (2017) claimed that diversity effects on tree growth need to be assessed in the context of a focal tree's local competitive environment. Species with different life‐history strategies will respond to differently to neighborhood diversity due to their different ability to tolerate competition. When the intensity of competition is high, acquisitive species may not benefit from a more diverse neighborhood because of their relatively low competition tolerance. For conservative species, the opposite is true. Similarly, Jucker et al. (2014) compared the difference of the growth response to species mixing between light‐demanding (pine) and shade tolerance species (oaks). They found that the positive effect of species mixing on pine growth was severely reduced due to the strong competition for light with neighboring trees, resulting in less room for complementarity. As light availability increased, light‐demanding species could benefit more from competitive reduction through niche complementarity. Given that light‐demanding species are dominant in the study area (e.g., *Pinus koraiensis* and *Tilia amurensis*, see Ni et al., 2014), we infer that the negative effects of biodiversity in close‐range neighborhoods may be caused by the intense competition for light. As there are many possible species combination in the neighborhood, it is difficult to isolate the effects of species‐specific interactions. However, we believe that this is an important question for future investigations.

Positive (facilitation) and negative (competition) interactions often occur simultaneously. Net biodiversity effect is the combined result of facilitation and competition interactions in response to neighborhood biodiversity (Forrester & Bauhus, 2016). Therefore, the change of direction of biodiversity effects reflects the relative influences of competition and facilitation to some extent. A negative diversity effect suggests that competition is more prevalent than complementarity or facilitation at small scales (<9 m). With increasing scale, competition pressure may be reduced. Neighborhoods with more species living together are able to utilize resources more efficiently, which enhances productivity. Interestingly, the positive effects of species diversity showed up at scales greater than 9 m, which corresponds to the biggest canopy radius (8.92) in our plot. We infer that at smaller scales (<9 m) limited resource availability results in stronger competition, especially among neighbors under the canopy area of a focal tree or under overlapping canopies. Our results show that there are no simple answers. The effects of biodiversity on forest productivity are manifold requiring a better understanding of species interactions at different neighborhood scales.

By using an individual tree‐based method, we can control several confounding factors at the same time. Many studies have shown that tree size is a key factor influencing tree growth (Coomes & Allen, 2007; Uriarte et al., 2004). As a tree becomes larger, its defense against environmental stress may improve. The productivity of structurally diverse, mixed forests is strongly influenced by structural diversity, which represented increased vertical stratification and crown plasticity (Dănescu, Albrecht, & Bauhus, 2016). In our study, the dominance of the focal tree in terms of DBH showed significant effects on tree growth, which suggests that in the analysis of the biodiversity‐productivity relationship, we should also pay attention to size structure variation (Ni et al., 2014; Pommerening & Grabarnik, 2019).

Chisholm et al. (2013) found that environmental gradients may drive specific pattern of the biodiversity‐productivity relationship. Duffy, Godwin, and Cardinale (2017) found that increases in biomass with increasing biodiversity, after controlling for environmental covariates, are larger in nature than has previously been reported. In our study, elevation was positively correlated with tree growth. This result is inconsistent with a study conducted by King, Gugerli, Fonti, and Frank (2013) which found lower growth rates at higher elevations. The difference between the two studies may be partially caused by the different spatial variations in elevation. The elevation gradient in our study is small (200 m) compared with 900 m in their study area. Additionally, we found a significant effect of canopy openness on tree growth, suggesting that competition for light is a major determinant of tree growth (Jucker et al., 2014). The nonsignificant effects of soil variables may be the result of a homogeneous soil condition in our plot.

Research into the relationship between biodiversity and ecosystem functioning is motivated by both a basic interest in understanding ecological communities and a practical need to conserve and manage forest ecosystem services (Chisholm et al., 2013; Liang et al., 2016). Woody productivity and biomass carbon storage are two key forest ecosystem functions. To meet the wide range of forest management objectives, which includes carbon sequestration and biodiversity conservation, the results of our findings may help to motivate and facilitate the transition of monocultures to mixed‐species forest communities.

## 5 CONCLUSION

Considering effects of tree size, abiotic condition, and neighborhood competition, species diversity is significantly correlated with tree growth, but these effects change with the spatial scale. Diversity effects were regulated by intense competition resulting in significantly negative diversity‐productivity relations at small neighborhood scales. Tree growth increases when there is reduced competition and greater tree species diversity in the neighborhood due to niche complementarity and facilitation at the scales larger than 9 m. We conclude that the use of individual tree‐based methods, as applied in this study, may lead to a better understanding of the biodiversity‐productivity relationship in multi‐species forest communities.

## CONFLICTS OF INTEREST

The authors declare no conflict of interest.

## AUTHOR CONTRIBUTION

**Chunyu Fan:** Conceptualization (lead); Formal analysis (lead); Investigation (equal); Methodology (lead); Software (lead); Writing‐original draft (lead). **Lingzhao Tan:** Software (supporting). **Chunyu Zhang:** Funding acquisition (equal); Methodology (equal). **Xiuhai Zhao:** Conceptualization (equal); Funding acquisition (equal). **Lushuang Gao:** Funding acquisition (equal); Investigation (equal). **Klaus von Gadow:** Writing‐original draft (supporting).

## Supporting information

Appendix S1

## Data Availability

he data that support the findings of this study can be accessed on Figshare: https://doi.org/10.6084/m9.figshare.12464252.
