# Supplementary material for: Scale‐dependent effects of neighborhood biodiversity on individual tree productivity in a coniferous and broad‐leaved mixed forest in China
Source: Ecol Evol. 2020 Jul 8;10(15):8225–34. doi: 10.1002/ece3.6530 (PMC7417230; doi:10.1002/ece3.6530)
Supplement: Supplementary file 1 — Appendix S1 [file ECE3-10-8225-s001.docx]

**APPENDIX**

**Table S1.** Basic information of the 29 species in our study area

| **Species** | **Genus** | **Family** | **No.individuals** | **Mean DBH**  **(cm)** |
| --- | --- | --- | --- | --- |
| *Betula platyphylla* | *Betula* | *Betulaceae* | 8 | 23.0 |
| *Acer mandshuricum* | *Acer* | *Sapindaceae* | 1843 | 13.5 |
| *Syinga reticulata var. mandshurica* | *Syringa* | *Oleaceae* | 691 | 7.9 |
| *Prunus padus* | *Prunus* | *Rosaceae* | 102 | 7.5 |
| *Ulmus davidiana var. japonica* | *Ulmus* | *Ulmaceae* | 31 | 15.7 |
| *Acer barbinerve* | *Acer* | *Sapindaceae* | 292 | 6.6 |
| *Ulmus macrocarpa* | *Ulmus* | *Ulmaceae* | 116 | 21.2 |
| *Betula costata* | *Betula* | *Betulaceae* | 576 | 29.1 |
| *Prunus maximowiczii* | *Prunus* | *Rosaceae* | 83 | 11.0 |
| *Pinus koraiensis* | *Pinus* | *Pinaceae* | 552 | 34.6 |
| *Juglans mandshurica* | *Juglans* | *Juglandaceae* | 681 | 27.2 |
| *Fraxinus rhynchophylla* | *Fraxinus* | *Oleaceae* | 8 | 25.1 |
| *Maackia amurensis* | *Maackia* | *Leguminosae* | 5 | 7.7 |
| *Phellodendron amurense* | *phellodendron* | *Rutaceae* | 634 | 23.4 |
| *Tilia mandshurica* | *Tilia* | *Malvaceae* | 70 | 25.1 |
| *Ulmus laciniata* | *Ulmus* | *Ulmaceae* | 1644 | 24.1 |
| *Corylus mandshurica* | *Corylus* | *Betulaceae* | 13 | 7.1 |
| *Quercus mongolica* | *Quercus* | *Fagaceae* | 124 | 36.6 |
| *Acer triflorum* | *Acer* | *Sapindaceae* | 1 | 62.6 |
| *Carpinus cordata* | *Carpinus* | *Betulaceae* | 3135 | 10.8 |
| *Acer tegmentosum* | *Acer* | *Sapindaceae* | 931 | 12.0 |
| *Acer mono* | *Acer* | *Sapindaceae* | 1875 | 20.2 |
| *Abies holophylla* | *Abies* | *Pinaceae* | 297 | 25.0 |
| *Malus baccata* | *Malus* | *Rosaceae* | 3 | 10.3 |
| *Populus davidiana* | *Populus* | *Salicaceae* | 8 | 32.1 |
| *Rhamnus davarica* | *Rhamnus* | *Rhamnaceae* | 16 | 10.5 |
| *Fraxinus mandshurica* | *Fraxinus* | *Oleaceae* | 291 | 20.1 |
| *Sorbus alnifolia* | *Sorbus* | *Rosaceae* | 659 | 15.9 |
| *Tilia amurensis* | *Tilia* | *Malvaceae* | 1371 | 25.6 |
| **Total** |  |  | 16060 |  |

**Detailed description of** **allometric models**

In August 2012, altogether 144 trees of 13 tree species were cut at ground level in Jiaohe. For each tree species, at least ten trees with different diameters were selected for a destructive measurement of aboveground dry biomass. Branches and leaves were removed first, followed by the measurement and recording of the diameter at breast height (*dbh*) and tree height (*H*). The whole stem was divided into several parts and dried to a constant mass at 85℃, and then the aboveground coarse woody biomass was weighed to the nearest milligram.

**Table S2.** The number and information of sample tree species

| Tree species | Number of samples | Range of tree dbh's/cm | Range of tree heights/m |
| --- | --- | --- | --- |
| *Abies nephrolepis* | 21 | 3.4-35.6 | 2.7-22.1 |
| *Acer mandshuricum* | 10 | 7.8-35.9 | 9.1-18.5 |
| *Acer mono* | 12 | 6.4-45.3 | 8.5-20.6 |
| *Betula platyphylla* | 10 | 5.7-40.0 | 9.3-22.8 |
| *Carpinus cordata* | 10 | 5.1-13.4 | 7.9-11.9 |
| *Fraxinus mandshurica* | 10 | 10.7-41.4 | 10.9-23.7 |
| *Juglans mandshurica* | 10 | 6.5-42.5 | 8.2-23 |
| *Maackia amurensis* | 10 | 4.9-25.4 | 7.0-18.2 |
| *Pinus koraiensis* | 11 | 8.4-44.0 | 6.7-22.3 |
| *Populus ussuriensis* | 10 | 9.1-47.1 | 10.5-26.4 |
| *Quercus mongolica* | 10 | 4.2-41.2 | 5.5-22.8 |
| *Tilia amurensis* | 10 | 7.0-42.2 | 9.6-22.5 |
| *Ulmus japonica* | 10 | 5.6-39.0 | 6.8-20.1 |

**Table S3.** Regression coefficients for the different equations used to estimate aboveground wood biomass. The allometric equation is AGB=exp(*a*+*b**ln(*dbh*)), where AGB is the aboveground coarse woody biomass (in kg), and *dbh* (in cm) is the diameter at breast height.

| Tree species | Fitting coefficients | | *R*^2^ | *RMSE* | Similar species |
| --- | --- | --- | --- | --- | --- |
|  | a (*SE*) | b (*SE*) |  |  |  |
| *Abies holophylla* | -2.989 (0.148)^***^ | 2.613 (0.057)^***^ | 0.991 | 0.164 |  |
| *Acer mandshuricum* | -2.111 (0.278)^***^ | 2.310(0.092)^***^ | 0.9875 | 0.1180 | *Acer barbinerve*; *Acertriflorum*;  *Acer tegmentosum* |
| *Acer mono* | -2.164 (0.175)^***^ | 2.336(0.056)^***^ | 0.9943 | 0.0982 |  |
| *Betula platyphylla* | -1.941 (0.170)^***^ | 2.286(0.056)^***^ | 0.9952 | 0.0908 | *Betula costata* |
| *Carpinus cordata* | -1.909 (0.440)^**^ | 2.111(0.197)^***^ | 0.9424 | 0.1562 |  |
| *Fraxinus mandshurica* | -2.301 (0.242)^***^ | 2.443(0.077)^***^ | 0.9921 | 0.1004 | *Fraxinus rhynchophylla*; *Phellodendron amurense* |
| *Juglans mandshurica* | -2.466 (0.280)^***^ | 2.381(0.091)^**^* | 0.9885 | 0.1494 |  |
| *Maackia amurensis* | -2.001 (0.256)^***^ | 2.198(0.101)^***^ | 0.9835 | 0.1428 | *Syringa reticulata* var. *amurensis*; *Prunus padus;* *Prunus maximowiczii; Malus baccata; Rhamnus davarica; Corylus mandshurica* |
| *Pinus koraiensis* | -3.394 (0.245)^***^ | 2.582(0.079)^***^ | 0.9917 | 0.1325 |  |
| *Populus davidiana* | -2.507 (0.233)^***^ | 2.358(0.072)^***^ | 0.9925 | 0.1075 |  |
| *Quercus mongolica* | -2.797 (0.386)^***^ | 2.571(0.128)^***^ | 0.9804 | 0.2483 |  |
| *Tilia amurensis* | -2.364 (0.391)^***^ | 2.323(0.126)^***^ | 0.9770 | 0.2039 | *Tilia mandschurica* |
| *Ulmus japonica* | -2.058 (0.339)^***^ | 2.271(0.112)^***^ | 0.9832 | 0.1923 | *Ulmus macrocarpa*;  *Ulmus laciniata*;  *Sorbus alnifolia* |

*SE*: The standard error; *R*^2^: The coefficient of determination; *RMSE*: The root mean square error; * indicates *p*< 0.05, ** indicates *p*< 0.01, *** indicates *p* < 0.001.

**Table S4.** Functional traits used in this study

| Functional traits | Mean | SD |
| --- | --- | --- |
| maximum height(m) | 16.9 | 5 |
| wood density(mg mm^-3^) | 0.48 | 0.09 |
| Leaf area (mm^2^) | 89.4 | 141 |
| Specific leaf area (mm^2^ g^-1^) | 271.2 | 73.9 |
| Leaf carbon concentration (mg g^-1^) | 46.2 | 2.4 |
| Leaf nitrogen concentration (mg g^-1^) | 2.41 | 0.49 |
| Leaf carbon-nitrogen ratio | 20.1 | 5.3 |

**Table S5.** Soil variable loadings on the PCAs

| Soil variables |  | OGF | | | | |
| --- | --- | --- | --- | --- | --- | --- |
|  |  | PC1 | PC2 | PC3 | PC4 | PC5 |
| Total nitrogen |  | 0.54 | 0.27 | 0.15 | 0.23 | - |
| Total phosphorus |  | - | -0.54 | -0.26 | 0.12 | 0.52 |
| Total potassium |  | - | - | 0.85 | -0.31 | 0.38 |
| Organic matter |  | 0.59 | 0.14 | - | 0.21 | - |
| Available nitrogen |  | 0.51 | -0.12 | -0.14 | -0.24 | -0.19 |
| Available phosphorus |  | 0.23 | -0.42 | -0.11 | -0.65 | -0.26 |
| Available potassium |  | - | -0.38 | 0.38 | 0.41 | -0.66 |
| PH |  | 0.18 | -0.52 | - | 0.38 | 0.20 |
| Proportion of Variance |  | 0.27 | 0.17 | 0.13 | 0.12 | 0.12 |
| Cumulative Proportion |  | 0.27 | 0.44 | 0.57 | 0.69 | 0.81 |

Means the term was not included in the PCA axis.

**Table S6.** Model comparison results of general linear models for selecting significant control variables

| Models (Only for control variables) | df | Log-Likehood | AICc | Delta | Weight |
| --- | --- | --- | --- | --- | --- |
| **~DBH+Elevation+Canopy+Hegyi+U** | **6** | **-21277.06** | **42574.1** | **0** | **0.68** |
| ~DBH+Elevation+Canopy+Hegyi+U+Aspect | 7 | -21277.76 | 42577.5 | 3.41 | 0.12 |
| ~DBH+Elevation+Canopy+Hegyi+U+Slope | 7 | -21278.00 | 42578.0 | 3.89 | 0.05 |

**Table S7**. Estimates of the control variables in species richness model at 1, 2, 3 and 4m scale.

| Control Variables | Coefficients  1m | Coefficients  2m | Coefficients  3m | Coefficients  4m |
| --- | --- | --- | --- | --- |
| DBH | 1.69^***^ | 1.69^***^ | 1.68^***^ | 1.67^***^ |
| Dominance index(U) | 0.24^***^ | 0.24^***^ | 0.23^***^ | 0.22^***^ |
| Elevation | 0.59^***^ | 0.58^***^ | 0.57^***^ | 0.55^***^ |
| Canopy openness | 0.18^***^ | 0.18^***^ | 0.17^***^ | 0.16^***^ |
| SR: Hegyi | 0.04 | -0.06 | -0.03 | -0.03 |

**Table S8.** Estimates of the control variables in species richness model at 6, 7, 8 and 9m scale.

| Control Variables | Coefficients  6m | Coefficients  7m | Coefficients  8m | Coefficients  9m |
| --- | --- | --- | --- | --- |
| DBH | 1.64^***^ | 1.63^***^ | 1.61^***^ | 1.60^***^ |
| Dominance index(U) | 0.21^***^ | 0.21^***^ | 0.21^***^ | 0.21^***^ |
| Elevation | 0.52^***^ | 0.52^***^ | 0.53^***^ | 0.54^***^ |
| Canopy openness | 0.16^***^ | 0.16^***^ | 0.15^***^ | 0.15^***^ |
| SR: Hegyi | 0.06^***^ | 0.08^***^ | -0.07^***^ | -0.11^***^ |

**Table S9.** Estimates of the control variables in species richness model at 11, 12, 13 and 14m scale.

| Control Variables | Coefficients  11m | Coefficients  12m | Coefficients  13m | Coefficients  14m |
| --- | --- | --- | --- | --- |
| DBH | 1.57^***^ | 1.56^***^ | 1.56^***^ | 1.55^***^ |
| Hegyi index | 0.14 | 0.07 | 0.09 | 0.09^*^ |
| Dominance index(U) | 0.20^***^ | 0.20^***^ | 0.21^***^ | 0.21^***^ |
| Elevation | 0.55^***^ | 0.55^***^ | 0.55^***^ | 0.55^***^ |
| Canopy openness | 0.15^***^ | 0.16^***^ | 0.15^***^ | 0.15^***^ |
| SR: Hegyi | -0.13^***^ | -0.10^***^ | -0.11^***^ | -0.11^***^ |

**Table S10.** Estimates of the control variables in species richness model at 16, 17, 18 and 19m scale.

| Control Variables | Coefficients  16m | Coefficients  17m | Coefficients  18m | Coefficients  19m |
| --- | --- | --- | --- | --- |
| DBH | 1.54^***^ | 1.53^***^ | 1.53^***^ | 1.52^***^ |
| Dominance index(U) | 0.21^***^ | 0.21^***^ | 0.21^***^ | 0.21^***^ |
| Elevation | 0.55^***^ | 0.55^***^ | 0.55^***^ | 0.54^***^ |
| Canopy openness | 0.15^***^ | 0.15^***^ | 0.14^***^ | 0.14^***^ |
| SR: Hegyi | -0.15^***^ | -0.15^***^ | -0.19^***^ | -0.21^***^ |

**Table S11.** Estimates of the control variables in Shannon index model at 1, 2, 3 and 4m scale.

| Control Variables | Coefficients  1m | Coefficients  2m | Coefficients  3m | Coefficients  4m |
| --- | --- | --- | --- | --- |
| DBH | 1.69^***^ | 1.69^***^ | 1.68^***^ | 1.67^***^ |
| Dominance index(U) | 0.24^***^ | 0.24^***^ | 0.23^***^ | 0.22^***^ |
| Elevation | 0.59^***^ | 0.58^***^ | 0.57^***^ | 0.55^***^ |
| Canopy openness | 0.18^***^ | 0.18^***^ | 0.17^***^ | 0.16^***^ |
| Hs: Hegyi | 0.09 | 0.06 | 0.02 | 0.04 |

**Table S12**. Estimates of the control variables in Shannon index model at 6, 7, 8 and 9m scale.

| Control Variables | Coefficients  6m | Coefficients  7m | Coefficients  8m | Coefficients  9m |
| --- | --- | --- | --- | --- |
| DBH | 1.64^***^ | 1.63^***^ | 1.61^***^ | 1.60^***^ |
| Dominance index(U) | 0.21^***^ | 0.21^***^ | 0.21^***^ | 0.21^***^ |
| Elevation | 0.53^***^ | 0.52^***^ | 0.53^***^ | 0.54^***^ |
| Canopy openness | 0.16^***^ | 0.16^***^ | 0.16^***^ | 0.15^***^ |
| Hs: Hegyi | -0.11^***^ | -0.16^***^ | -0.17^***^ | -0.22^***^ |

**Table S13.** Estimates of the control variables in Shannon index model at 11, 12, 13 and 14m scale.

| Control Variables | Coefficients  11m | Coefficients  12m | Coefficients  13m | Coefficients  14m |
| --- | --- | --- | --- | --- |
| DBH | 1.57^***^ | 1.56^***^ | 1.56^***^ | 1.55^***^ |
| Dominance index(U) | 0.20^***^ | 0.20^***^ | 0.21^***^ | 0.21^***^ |
| Elevation | 0.55^***^ | 0.55^***^ | 0.55^***^ | 0.55^***^ |
| Canopy openness | 0.15^***^ | 0.16^***^ | 0.15^***^ | 0.15^***^ |
| Hs: Hegyi | -0.24^***^ | -0.17^***^ | -0.17^***^ | -0.15^***^ |

**Table S14.** Estimates of the control variables in Shannon index model at 16, 17, 18 and 19m scale.

| Control Variables | Coefficients  16m | Coefficients  17m | Coefficients  18m | Coefficients  19m |
| --- | --- | --- | --- | --- |
| DBH | 1.54^***^ | 1.53^***^ | 1.53^***^ | 1.52^***^ |
| Dominance index(U) | 0.21^***^ | 0.21^***^ | 0.21^***^ | 0.21^***^ |
| Elevation | 0.55^***^ | 0.55^***^ | 0.55^***^ | 0.54^***^ |
| Canopy openness | 0.15^***^ | 0.15^***^ | 0.14^***^ | 0.14^***^ |
| Hs: Hegyi | -0.18^**^ | -0.16^**^ | -0.17^*^ | -0.17^***^ |

**Table S15.** Estimates of the control variables in functional diversity model at 1, 2, 3 and 4m scale.

| Control Variables | Coefficients  1m | Coefficients  2m | Coefficients  3m | Coefficients  4m |
| --- | --- | --- | --- | --- |
| DBH | 1.84^***^ | 1.75^***^ | 1.71^***^ | 1.69^***^ |
| Dominance index(U) | 0.11^***^ | 0.28^***^ | 0.26^***^ | 0.24^***^ |
| Elevation | 0.86^***^ | 0.68^***^ | 0.67^***^ | 0.57^***^ |
| Canopy openness | 0.12^***^ | 0.15^***^ | 0.14^***^ | 0.17^***^ |
| Hegyi index | -0.01 | -0.01 | -0.01 | -0.04^***^ |

**Table S16.** Estimates of the control variables in functional diversity model at 6, 7, 8 and 9m scale.

| Control Variables | Coefficients  6m | Coefficients  7m | Coefficients  8m | Coefficients  9m |
| --- | --- | --- | --- | --- |
| DBH | 1.64^***^ | 1.62^***^ | 1.61^***^ | 1.60^***^ |
| Dominance index(U) | 0.21^***^ | 0.21^***^ | 0.20^***^ | 0.20^***^ |
| Elevation | 0.56^***^ | 0.56^***^ | 0.56^***^ | 0.56^***^ |
| Canopy openness | 0.16^***^ | 0.16^***^ | 0.16^***^ | 0.16^***^ |
| Hegyi index | -0.09^***^ | -0.10^***^ | -0.12^***^ | -0.14^***^ |

**Table S17.** Estimates of the control variables in functional diversity model at 11, 12, 13 and 14m scale.

| Control Variables | Coefficients  11m | Coefficients  12m | Coefficients  13m | Coefficients  14m |
| --- | --- | --- | --- | --- |
| DBH | 1.58^***^ | 1.57^***^ | 1.56^***^ | 1.55^***^ |
| Dominance index(U) | 0.20^***^ | 0.21^***^ | 0.21^***^ | -0.21^***^ |
| Elevation | 0.56^***^ | 0.56^***^ | 0.56^***^ | 0.55^***^ |
| Canopy openness | 0.16^***^ | 0.16^***^ | 0.15^***^ | 0.15^***^ |
| Hegyi index | -0.15^***^ | -0.16^***^ | -0.16^***^ | -0.17^***^ |

**Table S18.** Estimates of the control variables in functional diversity model at 16, 17, 18 and 19m scale.

| Control Variables | Coefficients  16m | Coefficients  17m | Coefficients  18m | Coefficients  19m |
| --- | --- | --- | --- | --- |
| DBH | 1.54^***^ | 1.53^***^ | 1.54^***^ | 1.53^***^ |
| Dominance index(U) | 0.21^***^ | 0.21^***^ | 0.21^***^ | 0.21^***^ |
| Elevation | 0.56^***^ | 0.56^***^ | 0.57^***^ | 0.56^***^ |
| Canopy openness | 0.15^***^ | 0.15^***^ | 0.15^***^ | 0.15^***^ |
| Hegyi index | -0.18^***^ | -0.18^***^ | -0.18^***^ | -0.19^***^ |


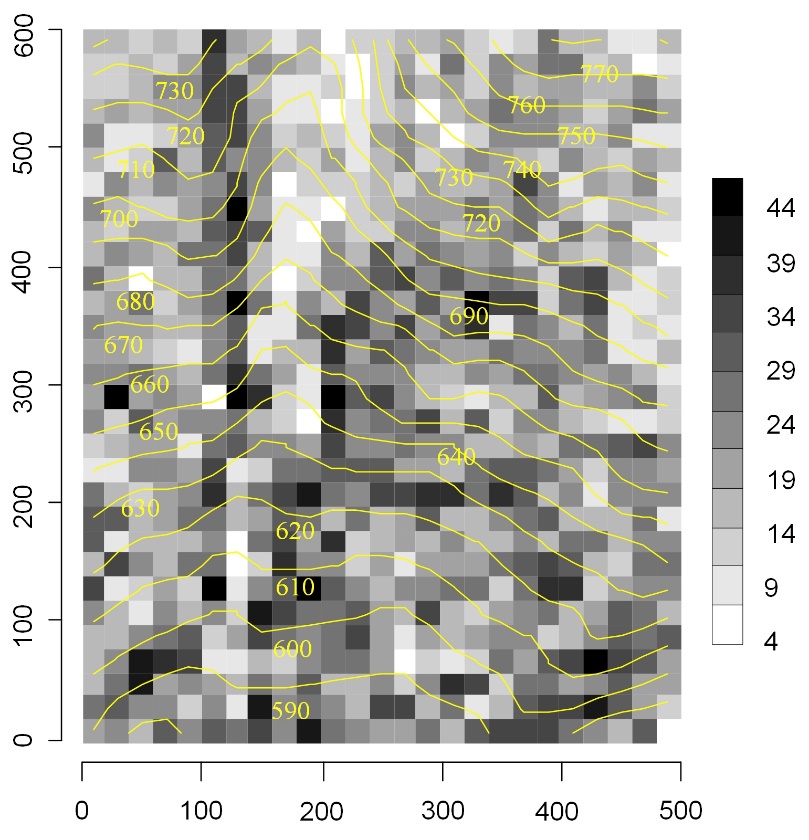


**Figure S1.** Topography and spatial distribution of 16060 individual trees in our plot at 20 m×20 m resolution.


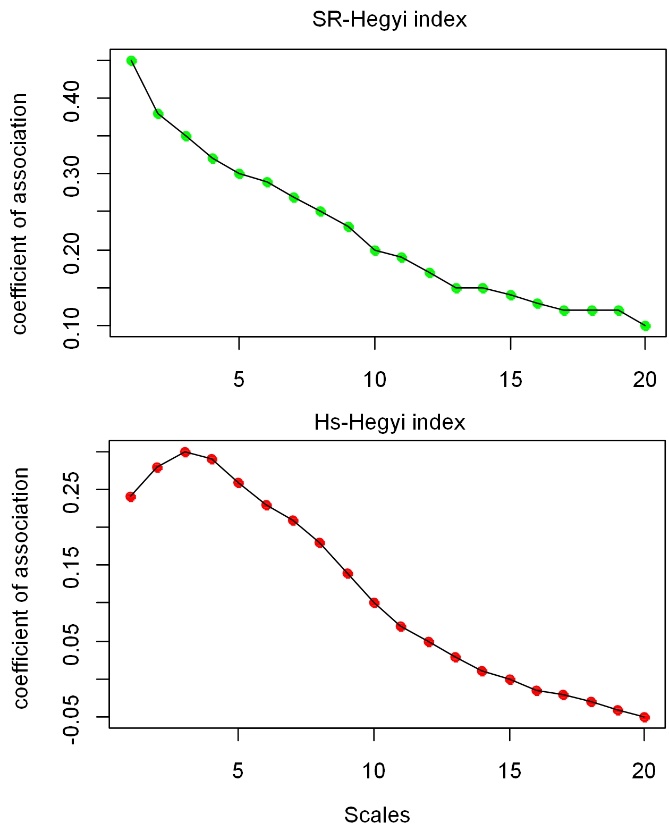


**Figure S2.** Correlation coefficients(p<0.001) between species diversity and competition index.
